# Impact of COVID‐19 on health and safety in the construction sector

**DOI:** 10.1002/hfm.20882

**Published:** 2021-01-12

**Authors:** Shelly Stiles, David Golightly, Brendan Ryan

**Affiliations:** ^1^ Gateway Consultants (HSW) Ltd. Burton‐on‐Trent UK; ^2^ School of Engineering Newcastle University Newcastle UK; ^3^ Faculty of Engineering University of Nottingham Nottingham UK

**Keywords:** construction, COVID‐19, health and safety, human factors, risk

## Abstract

Construction has been significantly affected by COVID‐19 yet is critical to the post‐COVID economic recovery. Specifically, construction needs to be constantly aware of safety and risk balanced with timely project delivery. Guidance for COVID‐19 must therefore be implemented in a way that reflects working practice and pressures. There is, however, a potential knowledge gap regarding the practical feasibility and impact of applying COVID‐19 measures within construction, made more difficult by factors such as the temporary nature of projects and complex working arrangements. This article presents a commentary on safe construction during, and beyond, COVID‐19, covering the human factors challenges and practicalities of implementing COVID‐19 measures. We observe that while guidance is strong on risk management, understanding of how best to implement this guidance is not yet stable. Also, care must be taken that implementing guidance does not detract from general safety, which is also challenged by increased pressures on delivery arising from COVID‐19. There may, however, be opportunities for safer working practice arising from new awareness of health, hygiene, and safety risk. The role of safety leadership is overlooked in guidance yet is vital to ensure safe application of COVID‐19 working practices. The key message is that COVID‐19 needs to be integrated and promoted within a general risk management approach, in part because this takes account of differing priorities regarding safety risks, rather than overly focussing on COVID‐19, and also because the effectiveness of COVID‐19 mitigations can be amplified by integration with pre‐existing safety processes.

## 1 INTRODUCTION

Construction is key to the global economy. In the United Kingdom, construction is worth over £100bn and employs over 2.4 million people (Rhodes, 2019). It is a sector that has seen a significant impact to its operations and has been amongst the hardest hit in terms of COVID‐19 (Koh, 2020; McClure et al., 2020; ONS, 2020). The implications of COVID‐19 on construction are twofold.

On one hand, work has been halted or changed and new projects paused while construction practices come to terms with new ways of working. Sites have had to adjust to social distancing, implementing new hygiene and personal protective equipment (PPE) measures, and accommodating a greater level of working from home for roles that are not essential to front‐line work. The importance of health and hygiene, as well as safety, has never been clearer. All of this has had to occur while maintaining safety in the conventional aspects of work, in a sector which ordinarily has multiple hazards. Delivering safety is a significant challenge (Health and Safety Executive [HSE], 2019; van der Molen et al., 2018), especially where multiple organizations of different sizes work together, as is typically found in medium to large construction projects (Peñaloza et al., 2020; Rowlinson, 2004; Stiles et al., 2012; Woolley et al., 2020). The temporary nature of arrangements can present a challenge for safety leadership (Stiles et al., 2018a), which is a key mechanism for engaging the workforce in safety (Zohar, 2002; Zohar & Luria, 2003).

On the other hand, construction is seen as a vital part of stimulating the post‐COVID economy, and there is much impetus to start work on “shovel ready” schemes (e.g., UK Gov, 2020). As an example, transport civil engineering is a significant part of this sector, with rail electrification and high‐speed rail seen as essential strategies to support decarbonization (International Energy Agency, 2019). In the more immediate term, short‐ and medium‐sized engineering projects are essential to maintain the road and rail network, down to local schemes to convert roads to cycle lanes, in a move to address new travel patterns arising from COVID‐19 (Laverty et al., 2020).

There is therefore significant need to ensure construction can quickly return to working safely, and in a flexible manner that might withstand subsequent local lockdowns, future waves or even future pandemics. In the U.K. construction sector, guidelines have been issued by bodies such as Construction Leadership Council (CLC, 2020), as well as guidance from the England and Wales Government (HM Gov, 2020) and HSE (2020). The question is therefore one of how well these guidelines address practice on the ground. Specifically:‐

- How well does guidance fit with the practicalities of safe working in construction? How successfully are construction sites implementing the guidance?
- How does COVID‐19, and associated guidance, affect the normal safety behavior and safe operation of construction? Do COVID‐19 practices impede conventional safety, or offer any potentially unanticipated benefits?
- What are the implications of organizational structure for safe working in construction during COVID‐19?
- What are the implications for safety leadership in construction during COVID‐19? What role can safety leadership play, and how must it adapt to address COVID‐19?

The following paper presents a commentary on these questions, based on reflection on the guidance against previous work in construction (Stiles et al., 2012, 2018a, 2018b), and observation and experience of the first author—a practicing health and safety consultant working on delivering safety and leadership guidance and behavioral change in construction, including during the COVID‐19 period. The contributions of the article are in describing how construction is meeting the challenge of working safely within COVID‐19, and identifying what further work is needed to continue to deliver safety in construction during COVID‐19 and beyond. While this article is based on experience in the U.K. construction sector, we note that the importance of construction to the economy, and understanding of the potential impact of COVID‐19, is shared worldwide (e.g., Choudhari, 2020; McClure et al., 2020; OSHA, 2020).

## 2 THE CONSTRUCTION CONTEXT

In normal times, delivering safety in construction is a complex activity (Woolley et al., 2020). The nature of the work is inherently often hazardous (Haslam et al., 2005), involving manual handling (Antwi‐Afari et al., 2017; Hartmann & Fleischer, 2005; Paquet et al., 1999), working around plant, working at height or with difficult postures (Dutta et al., 2020), working with dangerous materials (Sauni et al., 2001; Snashall, 2005) or where there are hazards such as electricity (e.g., during rail maintenance or installation of overhead line electrification [Salguero‐Caparrós et al., 2019]). It is a sector where safety has reached a plateau that still accounts for a significant number of injuries, lost working days, and a fatality injury rate in Great Britain (1.31 per 100,000 workers) that is three times the all industry rate (HSE, 2019) and where evidence of effective safety interventions is scarce (van der Molen et al., 2018).

Construction‐related risks include more than the work performed on site. There is movement to and from site, including travel for people to get to work, possibly to and from communal lodgings. At the site, there needs to be facilities for eating, for toilets and rest areas, for storage of materials, and for storage of tools. There is a variability in the type of tasks performed—while much civil engineering work may be outside, building or refurbishment (e.g., plastering, electrical work) may be performed inside. Construction is also a sector where the risks of safety must be balanced within the context of production and the need to deliver. Perfection in safety needs to be traded off against factors such as cost, capacity, efficiency, and quality (Peñaloza et al., 2020; Wilson et al., 2009).

Construction is a sector that was amongst the first affected by COVID‐19 (Koh, 2020) and has experienced a high rate of infection (ONS, 2020). A number of factors may account for this. For example, the sector has a high proportion of males, an ageing workforce with over 40% over 40 years old, and significant numbers over 55 (ONS, 2018) and with a high number of Black, Asian and Minority Ethnic (BAME) workers, and a high degree of migrant workforce that have been particularly hard hit by COVID‐19 in preliminary statistics (McClure et al., 2020). Additionally, work with hazardous materials and exposure to potentially harmful conditions that impact the respiratory function (Sauni et al., 2001) may also mean greater risk of underlying health problems linked to COVID‐19. While data are limited and direct causal pathways are still not well understood, these factors may explain why this sector has experienced higher incidence of COVID‐19.

**Figure 1:**
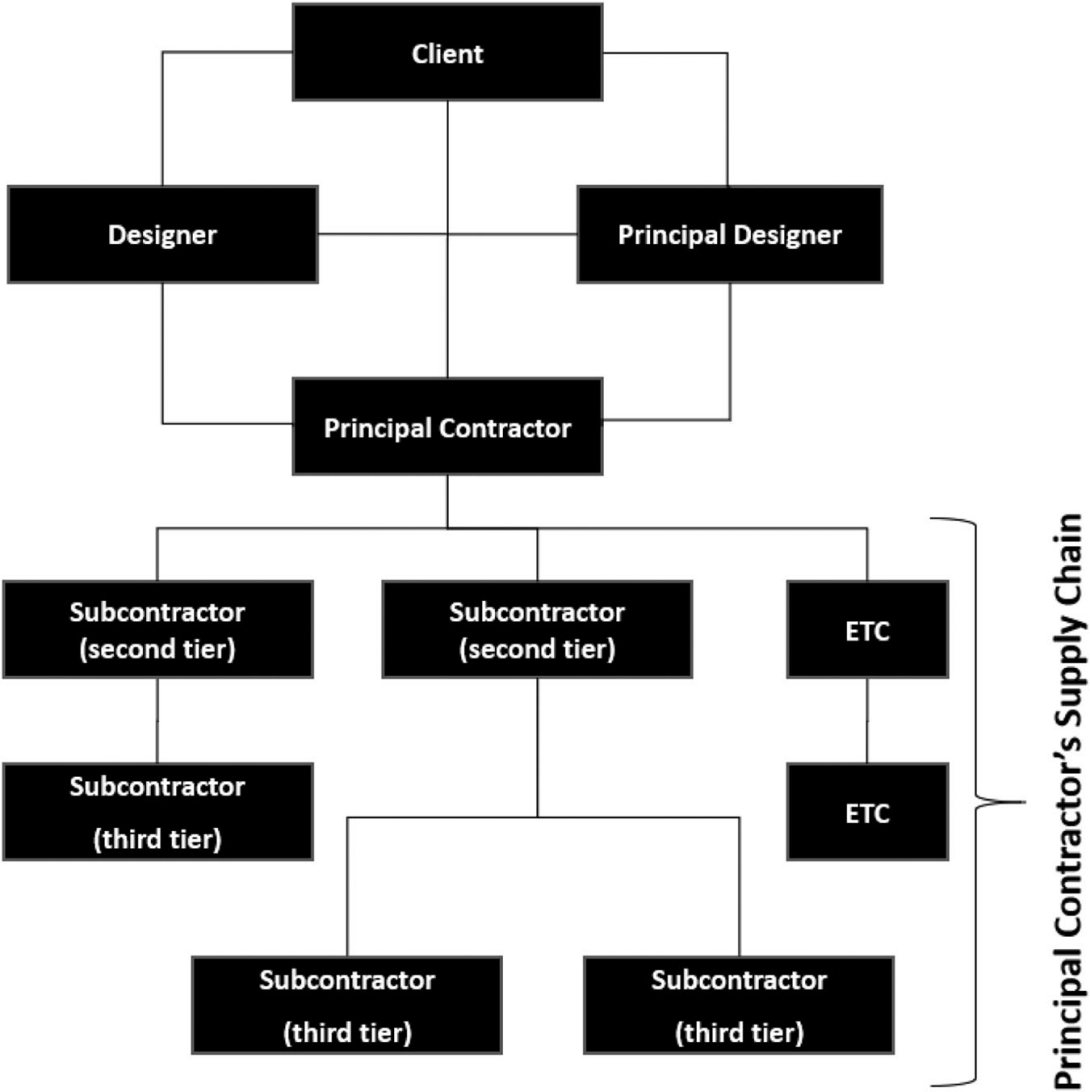
Typical structure of a Project Delivery Organization

One specific factor that impacts safety generally, and may have a bearing on COVID‐19, is the organization of work. Nearly all projects, particularly medium to large projects, are delivered through joint working of multiple organizations. A common project organization structure is developed, referred to as a “Project Delivery Organization” (PDO) for the remainder of this article, as illustrated in Figure 1. A PDO is established with a number of companies, co‐ordinated via contractual obligations, for a determined period of time (Rowlinson, 2004); key duty holders being the client, principal contractors, and the supply chain.

Table 1 presents an example of a medium‐sized PDO and some of the key characteristics (this is an anonymized aggregation of six pre‐COVID‐19 projects). These projects are highly dynamic, increasing in total numbers of people on site and numbers of sub‐contractors as the project progresses. Work is often delayed, so rather than being represented as a normal distribution, work and numbers of workers can be “skewed” to finish the job on time. Sub‐contractors may come from different organizations, often small (five people or less), and often engaged in specialist work (e.g., working at height, electrical installation, etc.).

**Table 1:** Typical medium‐sized Project Delivery Organization

Project scope	Civils/rail (e.g., suburban station construction)
**Project value (millions)**	£10 m
**Project duration**	52 weeks
**Number of site management team from PC on site**	5
**Number of people on site (average)**	40
**Number of people on site (peak)**	100
**Subcontracted workforce**	50%
**Number of accidents/incidents**	8

An implication of the PDO is that it can make more complex the communication and management of safety. Typically, safety is managed down the hierarchy, but feedback up the hierarchy is often restricted (Woolley et al., 2020). There may be different levels of safety culture (Stiles et al., 2018b) and variable application of safety processes within pockets of the PDO (Stiles et al., 2012). Companies employing fewer than 100 people have a tendency for higher accident rates, indicative of poor safety performance (Fairman & Yapp, 2005; Pinder et al., 2016; Vickers et al., 2003). Similarly, construction industry studies by the UK Health and Safety Executive showed that companies within this sector employing fewer than 400 people have significantly poorer safety performance than larger organizations (HSE, 2011). Projects and priorities change during their execution, with contractors leaving and joining, hampering clear management and communication of safety. Where there is a lack of commitment from the supply chain it is very difficult for the principal contractor to implement effective interventions due to the mixed messages received by the workforce regarding priorities (Briscoe & Dainty, 2005; Briscoe et al., 2001). It is therefore a relevant question as to whether the influencing factors for general safety within the PDO will also have a bearing on the implementation of COVID‐guidance.

One significant factor in the delivery of safety in general is leadership (Flin & Yule, 2004; Zohar, 2002; Zohar & Luria, 2003). Since COVID‐19 there has needed to be a rapid and wholesale change of construction site safety, health, and hygiene, relying on leaders to give clear direction and resources, as well as ensuring that the controls necessary to be COVID secure are in place across all construction sites. Effective safety performance is influenced from senior management where leaders should examine their own behaviors to become more effective leaders in safety. It is recognized that leadership drives culture, which in turn influences behavior (Zohar & Luria, 2003). However, the construction industry recognizes the lack of leadership across the sector (CIOB, 2008). Safety leadership is needed both in terms of senior management by driving safety and their visible commitment to safety (Marsh et al., 1998; Thompson et al., 1998), but also by front‐line supervisors, who demonstrate commitment by ensuring the workforce are involved in safety decision making, and prioritizing safety above production (Andriessen, 1978; Farrington‐Darby et al., 2005).

The implications of the PDO and leadership intersect with leadership responsibilities often unclear between the different organizations within the PDO (Arditi & Chotibhongs, 2005). While the principal contractor has the primary responsibility for co‐ordination of site safety, the employer also has statutory duties for their employees. Differences in safety culture and safety practice across the PDO may inhibit how safety leadership from the principal contractor influences the supply chain (Stiles et al., 2018a, 2018b).

### 2.1 COVID‐19 and guidance

It is important to consider the mechanisms of transmission of COVID‐19. Transmission is primarily airborne as droplets from person to person through face to face contact (e.g., coughing) (Fennelly, 2020; Setti et al., 2020), though recent evidence also indicates that the virus may be suspended in the air as aerosols for appreciable periods of time (Domingo et al., 2020). Carriers may be symptomatic or asymptomatic (Kenyon, 2020) and transmission is significantly greater in enclosed spaces. There is also a potential risk of spread of the virus through fomites (Stephens et al., 2019) on contact surfaces, with contamination by falling droplets or hand contact from a carrier. The virus can remain viable for different lengths of time, up to several days, depending on the surface material (Suman et al., 2020). Further transmission can occur via self‐inoculation, as the virus enters the body by the airways (nose and mouth), or the eyes, primarily by hand contact.

As a result, the global strategy has been one of limiting person to person contact, either through complete reduction in social contact (lockdowns, closing of workplaces, schools, public spaces, etc.) or through social distancing. This distance has varied by country and, over time, within countries. In England, the guidance at the time of writing is 2 m social distancing without mitigation, or 1 m with suitable mitigation (such as a face mask). Also, public health messaging has emphasized hygiene, particularly washing hands on a regular basis and cleaning and disinfection of contact surfaces.

This general advice has informed guidance for either work generally, or specifically for construction. Three relevant sets of guidance are the HM Government construction‐specific advice for construction and outdoor working, the Health and Safety Executive return to work advice, and the Construction Leadership Council COVID‐19 guidance. These are summarized in Table 2, with some examples of the content of the guidance in brackets. There have been several revisions of these documents and the summaries refer to the latest versions on 13/10/20.

**Table 2:** Summary of COVID‐19 guidance—general and specific to the construction sector

HM Government Construction‐specific guidance (version 10)
https://assets.publishing.service.gov.uk/media/5eb961bfe90e070834b6675f/working-safely-during-covid-19-construction-outdoors-240920.pdf
Thinking about risk (all need to engage in a risk assessment; consult with staff; failure to conduct COVID‐19 risk assessment, or to act on it, is a breach of health and safety law)
Who should go to work (consider whether needed on site; plan for minimum people; keep in‐touch with off‐site workers)
Social distancing (wherever possible, handwashing, different locations, different roles)
Customers, visitor, and contractors (managing contacts, providing/explaining guidance)
Cleaning the workplace (before opening, keeping the workplace clean, hygiene, changing rooms and showers, handling equipment, materials, waste) (cleaning procedures for shared equipment; handwashing)
Personal protective equipment (PPE) (should not encourage precautionary use)
Workforce management (shifts and breaks work travel, communications, and training)
Inbound and outbound goods (pick‐up and drop‐off; frequency; driver behavior)
Health and Safety Executive (as of 09/20)
https://www.hse.gov.uk/coronavirus/assets/docs/working-safely-guide.pdf
Talking with your workers (guide to communicating with staff, also consider if English is not first language)
Who should go to work (changing tasks to reduce risk; work from home if possible; if cannot work at home, protection, handwashing, minimum number of people)
Protect people at risk (plan for the vulnerable or with vulnerable family)
Getting into and leaving work (travel alone if possible, staggering arrival and departure times, handwashing)
Work area (social distancing, where you cannot distance, keeping work area clean)
Moving around work environment (only essential trips, restrict job rotation, temporary walkways)
Common areas (toilets, canteens, shower areas)
Good hygiene (handwashing, promoting hygiene, guidance for cleaning of hygiene areas)
Information and guidance (share information with workers, with visitors, hold conversations, listen and act)
PPE (personal protective equipment) (continue normal use)
Construction Leadership Council (England) (version 5)
https://www.constructionleadershipcouncil.co.uk/wp-content/uploads/2020/07/Site-Operating-Procedures-Version-5.pdf
When to travel to work (not when symptomatic; at higher risk; living in vulnerable group)
Travel to work (share with similar groups; good ventilation; pairing arrangements; if must use public transport avoid the peak)
Driving at work (travel between sites; share with same individuals; cleaning vehicle)
Site access and egress (one way systems; minimize congestion; hygiene; site inductions)
Handwashing (regular breaks; additional facilities; clean facilities)
Toilet facilities (restrict number at one time; clean)
Canteens and rest areas—(increase size of facilities; staggered break times)
Changing facilities (increase size; restrict number of people at one time)
Work planning to avoid close contact (with a hazard control approach)
Emergency service and first aid (plan; anticipate delays)
Cleaning (toilets; handrails; lift and hoist controls)

There are multiple factors to consider when looking at this guidance. First, there are significant similarities across the guidance. All have gone through several iterations to stay abreast of changes in science, national policy, and general awareness in society of COVID‐19 risk and mitigation. The guidance reflects approaches to reduce the primary modes of transmission (airborne as droplets and aerosols and through contact surfaces) and the major mitigations (social distancing, ventilation, hygiene/handwashing, cleaning of contact surfaces). All have, at least in part, a typical hierarchy of controls approach to managing COVID‐19 risk, including seeking to eliminate exposure by only working on site where necessary; reducing exposure through social distancing, cleaning, and hygiene; and then working through isolation, control, PPE and behaviors. It is only the CLC guidance, however, that explicitly structures the risks in accordance with a hierarchy of controls. Both the HM Government and HSE advice is structured more in terms of the functions being managed (e.g., the work area, getting to site, moving around and in between sites).

All sets of guidance highlight that the areas of risk extend beyond the core activities of construction to include travel, rest facilities, changing facilities, materials in and out, and so on. From an ergonomics standpoint, all emphasize the need to consider job re‐design to minimize exposure, including some quite specific guidance (e.g., from the HM Government guidance, to encourage working side‐by‐side rather than face‐to‐face). There is also an emphasis on organizational aspects (e.g., the design of shifts to minimize exposure). However, while all mention engagement with staff, this is not covered substantially.

## 3 OBSERVATION 1: MANAGING COVID‐19 RISK IN CONSTRUCTION

The first question to address is how the practice of construction work has adapted to working in a manner that manages the risk of COVID‐19. Relevant to that question is the issue of how guidance has been applied, and whether there are limitations or gaps in this guidance.

### 3.1 Implementing the guidance

Unlike other sectors (e.g., retail, hospitality) that experienced an almost complete shutdown for several months, many sites continued to work in some form throughout the peak of COVID‐19 in the United Kingdom, during March–June 2020. The guidance, particularly from CLC, HSE, and the UK Government was released quite quickly and then rapidly updated as either new knowledge came to light, or the severity of the outbreak in the United Kingdom eased. Construction is a sector that is able to respond to changes in circumstances and regularly deals with changes in health and safety guidance and regulation. While changes have been difficult, people and processes have been able to react swiftly. This has been helped by guidance being well‐publicized across the industry, and that, as a public health emergency, there has been a constant public and media attention on COVID‐19.

One of the challenges of working under COVID‐19 for construction has been the practical implementation of guidance, and knowing what is practicable when implementing procedures such as cleaning. An example is the cleaning of touch points around site infrastructure, such as ladders and scaffolding. There is clearly the potential for either very frequent or irregular usage in the course of construction work, posing questions of whether they should be cleaned after every use, hourly or daily? Staff and management alike are still interpreting how best to implement the general guidance so as to meet on‐site requirements. Interpreting the guidance extends beyond the core activities and functions of “building” to include supporting functions such as canteen and mess facilities, toilets, and site security.

Overall, while there is an awareness of new standards, it is not always clear how these should be implemented. The understanding of what is needed to cause infection is evolving and the application of these mitigations is unprecedented with no guidance to share examples of good practice. The fact that the Health and Safety Executive (the U.K. safety regulator) has powers, and new funding, to enforce COVID‐19 compliance can exacerbate anxieties over what constitutes appropriate measures.

### 3.2 Applying controls

Some functions and processes lend themselves more readily than others to mitigation based on how readily COVID‐19 risk management can be integrated into pre‐existing processes. For example, the guidance highlights the need for cleaning of tools and equipment. As a matter of standard practice on large projects, large plant (e.g., excavators) will have “(ignition) key control” processes, whereby only competent staff are able and required to sign the equipment out for use and to sign it back afterwards. It is therefore relatively straightforward to link this process to additional COVID‐19 relevant steps, such as cleaning the seat and controls before plant is handed back. On the other hand, smaller pieces of equipment (e.g., shovels) are usually taken from communal stores on an ad hoc basis without specific controls. These communal stores may not have a “stores person” to manage the tool cleaning, and so forth, and thus new COVID‐19 controls are more difficult to apply. Making arrangements for secure storage of such tools on site can increase contamination risk.

The example of using a stores person to manage communal tools highlights the choice between collective versus individual controls. While collective measures are higher up the control hierarchy, they often require extra resources (in cost, an extra person on site, management time, and surveillance). Individual control measures have greater reliance on individual compliance to COVID‐19 controls, as there is less reliance on organizational safety management processes. This is widely accepted as the less preferred option for risk mitigation and control (UK Gov, 1999).

Control measures may be linked into a compliance and assessment regime, but this needs provision of new roles and competencies to check compliance. The CLC guidance provides checklists to cover when to conduct cleaning, but this needs processes to manage, including consideration when checklists do not work and complacency sets in (Rydenfält et al., 2014).

### 3.3 Screening and testing

This brings on the question of what to do and what processes need to be in place should someone be later found to have COVID‐19. Processes for conducting prework “tests” such as temperature checks, questions at security, and induction around symptoms are all appearing. Given that there needs to be a log of people on site for fire regulation, and so forth, this is another example of being able to link into pre‐existing processes. However, the efficacy of such tests in detecting infection in pre‐ or a‐ symptomatic individuals is limiting (Mitra et al., 2020). Enhanced processes might involve random swab testing across the site, but would be prohibitive in terms of planning, cost, and availability of testing capacity. What is reasonably practicable is a question of judgment. That said, there is already a culture and expectation of Drugs and Alcohol testing, both at induction and spot checks, particularly for working on highways or rail. This may be particularly useful in future with widespread antibody testing and, eventually, vaccination.

### 3.4 Communication and engagement

Engagement is referenced within the literature (e.g., Conchie et al., 2013) and guidelines and, in practice, sites are finding new ways to accommodate engagement. There is a reliance on face to face briefings to deliver key safety information, and these are increasingly conducted outside, taking account of social distancing restrictions. Whilst the weather has on the whole been accommodating over summer months, how to carry out briefings externally as COVID‐19 continues into the winter months may need further consideration. Before COVID‐19 formal engagement with the frontline was often in meetings (safety committees or safety action groups) or through on‐site leadership tours. This has changed, particularly on larger projects, due to often limited internal space to accommodate face to face sessions whilst meeting social distancing requirements. With fewer non‐essential site visits being undertaken from those not permanently site based (to reduce risk of transmission and exposure) there have been fewer leadership tours undertaken. This form of engagement generally takes place in less confined areas with general dilution ventilation, and so does provide a means of continued leader and workforce engagement.

Since the start of the pandemic, there has been an increased use of technology to facilitate communication and engagement. Whether these are replacing traditional face to face meetings now hosted via an online system, or through increased use of social media channels set up within projects to communicate more effectively, and remotely, for example, whatsapp groups. Another potential use of technology is QR Codes to track individual briefing records within a project. Up to this year, these would have predominantly been captured via paper and pen signature of receipt. It is still early days to determine whether technology could be an enabler for improved safety practices, culture, and behaviors, noting that the construction sector is not renown for embracing technological solutions (Okpala et al., 2020) and there are potential implications for increase in stress and work‐life balance with implementation of new technology (Holden & Sunindijo, 2018). Also, we note that a high number of migrant workers in the construction sector leads to a language barrier that can impede safety messaging (Bust et al., 2008; Oswald et al., 2019).

A final comment is that the nature of the pandemic, affecting society as a whole, seems to be helping to push through change management, acceptance, and adoption of change at a much faster rate than normally experienced with construction health and safety initiatives. While research has established the link between taking work‐related safety back to home life (Anger et al., 2018; Fleming, 2001), this may be an example of safety in the home (and wider society) taking safety into the workplace.

## 4 OBSERVATION 2: BROADER IMPLICATIONS FOR SAFETY

The impact of COVID‐19 would also appear to have wider implications for safety in general in construction. These are both negative and positive.

### 4.1 Negative implications: COVID‐19 as a distraction

The observation from current working, is that some sites seem to be focussed on COVID risk at the expense of awareness and vigilance of more general safety. This is in part because COVID‐19 has acted as a distraction, reducing the capacity of front‐line workers and management to focus on day‐to‐day safety concerns. While there is no data as yet, the impression is that safety standards have slipped back and there is a lack of focus, particularly on those sites where safety behaviors and culture were already less than ideal.

There have also been observed instances where work changes to accommodate COVID‐19 guidance has introduced a secondary risk. For example, as a result of site rules to reduce the numbers of people using lifts and hoists at any one time, queues can form and workers elect to carry equipment up staircases with greater risk of falls or dropping tools.

### 4.2 Negative implications: Reduced resources

The need to reduce people on site has typically been applied to managerial roles. This includes safety management roles, where those who may have regularly been on‐site now rarely visit the site. The result is that there is a reduction in focus on general safety on site, resulting in reduced compliance. Additionally, an implication of COVID‐19 is that there have been fewer competent people on site, either through illness or due to efforts to limit the numbers of people for reasons of social distancing. The implication is that, with fewer competent people, staff have to resort to workarounds and less safe practices. For example, with fewer people on site with competencies for using plant to move materials and tools, workers may resort to manual lifting and carrying, and pressure remaining to work more quickly for those operating the plant.

### 4.3 Negative implications: General wellbeing

There is an unknown impact on general wellbeing of the workforce. Frontline staff, many who have worked through the peak of the lockdown, are working under time pressure, with reduced resources, in a high‐risk environment, with the added pressures of COVID‐19 working practice. This is achieved while dealing with household challenges that COVID‐19 has brought with it—ensuring elderly relatives are well, keeping on top of shopping, home schooling of children, and so forth. This combination of factors may well have a bearing on long‐term mental health in a sector where this is already an issue (Choudhari, 2020; Love et al., 2010).

### 4.4 Positive implications: Pushing the health and safety agenda

Despite the challenges of COVID‐19, there have been benefits for safety. The need to adapt to COVID‐19 has generated a readiness to change. The sector has been able to respond rapidly to health and safety‐related change, and has thus demonstrated to both management and front‐line staff what is achievable when priority is given to health and safety. This experience may well benefit safety promotion in the future.

Furthermore, while there is often an emphasis on the safety aspect of “health and safety,” and health and hygiene are often overlooked (Jones et al., 2019). COVID‐19 has presented an opportunity to emphasize the importance of more general hygiene practices as a major consideration for construction. This may give impetus to considering health and health‐related behavior change (see Mullan et al., 2015) beyond the COVID‐19 outbreak.

### 4.5 Positive implications: Work redesign

The need to re‐design work and processes in light of COVID‐19 guidance has also presented an opportunity for a more general work re‐design to improve safety. In particular, thinking of the hierarchy of controls, COVID‐19 has provided an impetus to think more about the circumstances where people can be kept at distance from dangerous equipment or activities. Safer behaviors may be developed by new prescribed practices by safety managers or supervisors, but COVID‐19 has also generated some ad hoc behaviors that are developed by the workforce. In a recent observation as an example, placing steelwork columns (e.g., for steel frames or for rail overhead line masts) onto bolts is usually a task where people can expose themselves to finger and hand entrapment by moving too close when positioning the steel. Instead, workers have taken to using metal rods to push the steels into place to maintain social distancing, but also reducing the overall safety risk of entrapment. There is the opportunity to observe and learn from these ad hoc workarounds and implement them in more routine practice.

## 5 OBSERVATION 3: ORGANIZATIONAL FACTORS

### 5.1 Organizational pressures

Many projects are still working to the same timelines and delivery dates expected of their clients, despite a drop in productivity during the peak of lockdown, and an ongoing reduction of site staff. This creates extra pressure to deliver, which again may be at the expense of general, and COVID‐19 specific, safety. Additionally, the final stages of a project are often where specific trades come in for “fit‐out” (e.g., electrical work, plastering). While normally trades work around each other in what can be rather confined spaces, social distancing makes these arrangements much more complex. Where construction involves buildings, these final project stages are more likely to occur indoors, with a higher risk of COVID‐19 transmission. The difficulty of these arrangements is exacerbated by these trades often being self‐employed or working for small companies within the supply chain. It is not simply that people within the same organization are having to coordinate, but that people from different organizations need to consult and coordinate in a way that has not previously been required. This requires extra time and resources, and in practice people may try to work round each other in an unplanned, and often risky, manner. One implication is that some sites are moving to 24‐h working to meet deadlines, creating both fatigue risks (Hallowell, 2010; Maynard et al., 2020) and issues around handover.

### 5.2 Impact of the PDO

While the guidance shows the path to implementing COVID‐19 mitigation, there is a question in a complex, fluid arrangement like the PDO of who is responsible. Typically, safety on site is the responsibility of the Principal Contractor. However, each organization has a role to play in ensuring health and safety. Having clear responsibilities is important to eliminate confusion (Arditi & Chotibhongs, 2005). For example, if a small and specialist company undertakes scaffolding erection for a principal contractor, they will be responsible for the providing clean (sanitized) equipment, and workers using cleaned tools and vehicles. The question of who is responsible for maintaining the appropriate level of hygiene for the completed scaffolding needs clarification, which before COVID‐19, any maintenance responsibility would have been with the Principal Contractor. With many contractors on site, often at the same time, lines of responsibility for the additional COVID‐19 controls can become blurred.

The size of the project would also appear to be a factor in COVID‐19 management. Larger sites, with major clients and principal contractors, may have a greater level of awareness and rigor in comparison to smaller sites. Project size may have both a cultural and pragmatic contribution to how safety is managed post‐COVID‐19. Larger sites are more likely to have processes for the use of large plant where key control processes can be adapted to include cleaning (see Section 3.2). Processes may have lower levels of compliance on a smaller project, which may only have one or two pieces of plant and these are always operated by the same competent workers. However, there is likely to be a reduced risk of transmission on smaller projects with fewer workers, than on larger projects.

### 5.3 Transient workforce

PDOs often include small organizations, or self‐employed workers, sometimes working to short or zero hours contracts. This can put a pressure on the small company or individual to be on site, to be paid, despite the personal risks and anxiety that might occur within the current pandemic. Contractors commonly work across multiple sites (e.g., plant operators and other specialists may be rapidly moving from one site to the next). This presents a risk of cross‐contamination of COVID‐19 across sites, as well as challenges for the individual or supplier to understand differences in working arrangements on sites working under different implementations and interpretations of the guidelines.

There may be limited commitment to safety if an individual or supply chain company is only on a project for a short period of time. Finally, as the national strategy of containment moves to one of local lockdowns, as has been the case, there is a risk that people who travel into the site from elsewhere will not be able to get to work. Again, this presents pressures for the individual and challenges for the PDO in co‐ordination and delivery of work, resource planning, which then feed through into pressures that conflict with general safety.

## 6 OBSERVATION 4: THE ROLE OF SAFETY LEADERSHIP

### 6.1 Mechanisms of leadership

As noted previously (see Section 3.4), one of the key elements of leadership is a presence on site and leaders being able to exhibit and reinforce safety behaviors (Stiles et al., 2018a). However, the restriction of people on site means that this is challenging during COVID‐19. It is too early to understand whether it shows commitment to safety by leaders staying away (i.e., following the same rules as everyone else), or weakens leadership and promotes a perception that they are not involved or fearful of coming to site.

More generally, it is not yet clear which leadership mechanisms (Stiles et al., 2018a) are still relevant when people are remote from the site. Safety leadership tours are a well‐established practice within the construction sector, focused on leader and frontline workforce engagement, however many of these have stopped taking place with leaders undertaking COVID Compliance audits rather than focusing on engagement with workers. Leaders (senior managers, senior project managers, senior safety staff) are instead having to look at other means to communicate with staff and raise the profile of safety—both in terms of COVID‐19 specific measures, and safety generally. Video conferencing has been adopted widely, but it is unclear yet how best to use this mechanism for maximum positive impact on frontline safety engagement, particularly given that construction is not a sector that is usually quick to adopt new working practices and technology (Okpala et al., 2020).

### 6.2 Leadership knowledge and skills

Effective leadership is more than a delivery issue. It is a question of making sure that leaders have the right skills and competencies to actively lead, particularly through the period of turbulence since COVID‐19. How best to implement guidance is still emerging, as is an understanding of what constitutes good onsite management, compliance monitoring, and enforcement in this type of crisis situation (Dirani et al., 2020). Until this is fully understood, it is not always clear to leaders what are the appropriate messages to communicate. Is it important to wholly focus on COVID‐19 controls as this is the new risk facing the population, and when is it appropriate to cascade routine health and safety messages, and by what medium? Finally, it is not yet clear what construction workers are expecting from their leaders to support them through COVID‐19. It would be valuable to systematically capture what they need from their leaders, and how they want them to communicate, given current working and challenges. Once there is a better understanding of these factors, it may be necessary to upskill and develop leaders within the sector to effectively lead safety within PDOs.

### 6.3 Intersection with the PDO

Again, we need to consider that a construction project is not a singular organization, but a temporary conglomeration of organizations of different sizes. In this context, there is a question of who should be the visible leaders that will have the most influence on the workers on the ground. Should it be from the client, the principal contractor, or from contractors? While this is always a relevant question (Stiles et al., 2018a), it is transformed by COVID‐19 because it is more difficult for leaders to be visible and be active. If, for example, only the leaders from the principal contractor are able to be visible on site, what should they be prioritizing? Also, what messages and how should the leaders within contractor organizations working on projects communicate to their employees, who are under the day to day management of a principal contractor? The situation raises the question about co‐ordination of communication, consistency of messages, how these are reinforced as well as opportunities for worker feedback. Safety leaders from both the principal contractor and contractor (as employer) must continue to reinforce their messages in a manner that is meaningful to everyone under their management and supervision.

## 7 DISCUSSION

This article has reviewed the current state of working under COVID‐19 for the construction sector. Observations are summarized in Table 3, including recommendations and arising from this review and suggestions for future research. Looking across the observations, some key messages stand out. First, COVID‐19 is a risk like many others (Wilson et al., 2009), albeit one that has affected the industry on an unprecedented scale. It is likely that the most effective way of managing that risk is where it can be integrated into existing mechanisms of control. This both makes implementation easier and simplifies the workforce communication of the risk mitigation for COVID‐19. The observations are applicable to all organizations within the PDO, not just the principal contractor.

**Table 3:** Summary of observations, recommendations, and future research work

Observations	Sub‐themes	Recommendations	Future research
1. Managing COVID‐19 risk in construction	Implementing the guidance Applying controls Screening and testing Communication and engagement	Embed COVID‐19 controls within pre‐existing safety controls wherever possible Participatory input on task redesign New roles and competencies for COVID‐19 compliance Apply best practice from other sectors on use of communication technology	Defining effective measures of success for COVID‐19 control effectiveness Formal assessment of success of new communication technologies Development of communication best practice Understanding epidemiological risk in construction (and sub‐types of construction)
2. Broader implications for safety	Negative implications (COVID‐19 as a distraction; general wellbeing) Positive implications (pushing the health and safety agenda; work redesign)	Monitor and maintain levels of competence on‐site Exploit readiness to change	Data collection (e.g., accidents vs. infection) on the trade‐offs in COVID‐19 safety and overall site safety Exploration of effective co‐messaging of COVID‐19 and general safety
3. Organizational factors	Organizational pressures Impact of the PDO Transient workforce	Determine responsibilities, communication, and collaboration	Developing and validating risk trade‐off frameworks (e.g., Wilson et al., 2009) Establish guidance for safety collaboration across PDO
4. Role of safety leadership	Mechanisms of leadership Leadership knowledge and skills Intersection with the PDO	Leadership skill/competency development Maintain leadership visibility and commitment to safety	Determining effective safety leadership practices when leaders working remote from site Survey of front‐line staff to understand changing leadership needs Development of leadership competence to meet future needs

The second message is that we do not yet fully have to the tools to manage that risk. The guidance provides a strong framework, but there are still uncertainties about the implementation of the guidance both at an individual and organizational level. These tools include the need for evaluation studies to determine what are the effective ways of measuring and understanding whether methods are successful in managing COVID‐19 (Pawson et al., 2005; Pedersen et al., 2012). What is “reasonably practicable” is still emerging, and this is made more complex by COVID‐19 being a dynamic phenomenon (Rasmussen, 1997) both in terms of the science of transmission (Setti et al., 2020), the risk it represents (e.g., in terms of a “second wave”) and changing societal attitudes and perceptions. Organizations will have to learn to continue to operate with this degree of uncertainty for some time to come (Grote, 2004).

Third, the role and influence of the PDO goes beyond the practicalities of needing to brief people arriving on site. It has a whole range of implications both in terms of mitigation (e.g., how contractors will clean, use and store the tools they bring to site) but also communication and collaboration (e.g., how they understand the different arrangements in the different workplaces they attend; how multiple trades work together to coordinate safe working). This is a responsibility that is shared by the Principal Contractor and all of the supply chain. We note that individual measures are unlikely to have an effect on their own and it is likely a range of measures will need to be taken together if there is to be significant impact on safety (van der Molen et al., 2018).

The final message is that there are benefits and opportunities that will arise from dealing with COVID‐19 risks. Organizations have found that they can adapt rapidly, improve hygiene, redesign tasks, and adopt new technology. All of these are valuable lessons for safety professionals to draw upon for the ongoing challenge of COVID‐19 and for safety generally. However, there is a note of caution here. National governments are anticipating a construction‐led economic recovery from COVID‐19. This may be an appropriate vision, but not without some risk to safety, especially where many changes in work organization and practices are implemented with some urgency. We believe that findings from this article will give some direction to researchers, industry, and policy makers for steps that are needed to understand more about the correct balance between safety and construction performance.

There are a number of pieces of practical guidance that can be offered at this stage. The first, and following on from the above, is to try to implement COVID‐19 management within pre‐existing risk management where appropriate. This will likely lead to lower overheads and greater familiarity with the processes required. Organizations should look to develop their skills at virtual and video conferencing. For example, the medical sector has published guidance on how best to conduct conference calls (Oeppen et al., 2020) and this could be used, maybe with adaptation, for application in construction. Learning these skills will be vital given that the change to work and remote working is likely to continue after the end of COVID‐19.

**Figure 2:**
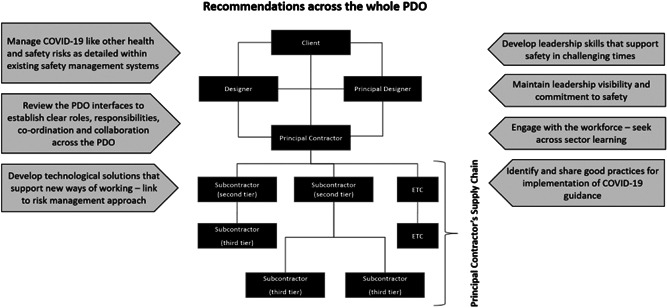
Recommendations for Project Delivery Organisations (PDOs)

Finally, as with all behavior change, it is important to engage the workforce as much as possible in the development of COVID‐19 policy and processes (de Jong & Vink, 2002; Wilson, 1995). This includes the participatory design of work (see Section 3.2) and consultation with construction workers and supervisors, to understand the practical experience and knowledge of what works and what does not, and to appreciate what skills, qualities, and means of engagement that they expect of their safety leaders (see Section 5.2). In the case of transient and contract workers, this is the knowledge that needs to be acquired across multiple sites and working conditions. These recommendations, and others, are mapped onto the PDO diagram (see Figure 2) and presented in Table 3.

Supporting COVID‐19 working in construction also requires answers to a number of research questions. The first of these is to understand what truly is the epidemiological impact of COVID‐19 on construction. While initially this was seen as a sector of risk, it is unclear whether the early indications of infections were due to a faster return to work in comparison to other sectors or some specific factor of construction work, or its demographic. Also, and possibly more relevant to those in human factors and ergonomics, is whether there was an overall decline or improvement in site safety, and in which categories of incidents or accidents changes occurred. This could be modeled using resilience frameworks (Peñaloza et al., 2020; Wilson et al., 2009) to understand the new parameters and trade‐offs involved. The role of the project safety hierarchy (Woolley et al., 2020) and, specifically, leaders in the management of COVID‐19 alongside other health and safety risks is worthy of further exploration. It is not yet known whether the tried and tested activities for demonstrating visible leadership and commitment to safety (Stiles et al., 2018a) are still relevant and impactful in this type of situation. Once there is greater clarity over whether COVID‐19 has changed what constitutes good safety leadership within a PDO, there will be a need to reassess the existing skills of leaders, bridging any skills gaps where necessary. There is a need to understand the future role of technology as an enabler, and potential necessity, for the facilitation of robust, open, and timely communication and engagement contributing to the management of risks and associated onsite safety performance.

Acknowledging the limitations of this article (U.K.‐based construction, medium scale projects, informal observation rather than empirical data and analysis), work should look at the multitude of different construction projects. This needs to cover the very small (e.g., domestic construction) through to the very large (projects like High Speed 2), and those with a high priority and value (e.g., rail electrification [DfT, 2020]). It should also cover specialist types of work, such as working in tunnels, underground, or other enclosed spaces, that might put particular pressures on adhering to guidance. This study should take a global perspective, recognizing the huge variation in factors that influence construction across the world, such as understanding the ramifications of migrant construction workers in places such as India and the Middle East (Buckley, 2012; Choudhari, 2020; Oswald et al., 2019).

Finally, relevant lessons could be shared from the construction industry with other transient sectors such as logistics and distribution, health services, that have similar reliance on multiagency collaboration and a disparate workforce (Winkler & Irwin, 2003); each of these continuing to work throughout the pandemic.
